# Editorial: Induction and Maintenance of Long-Term Immunological Memory Following Infection or Vaccination

**DOI:** 10.3389/fimmu.2019.02658

**Published:** 2019-11-08

**Authors:** Michael Vajdy

**Affiliations:** EpitoGenesis, Inc., Sacramento, CA, United States

**Keywords:** immunological memory, B cell memory, T cell memory, vaccination, adjuvant, infection

The term immunological memory is attributed to the phenomenon of qualitatively and quantitatively improved and/or enhanced antigen/epitope-specific recognition by various cells of the adaptive immune system with an ensuing improved and/or enhanced effector function. Because of the terminology used to describe this phenomenon, efforts have been focused on how to fit this phenomenon into various hypotheses, which all have to involve some form of remembrance of the previous interaction with a specific antigen/epitope. However, the way linguistics, language and words influence our thought process is an important area which was beyond the scope of this special issue.

How the adaptive leukocytes remember previous encounters has been mainly attributed to internal switching mechanisms involving prolonged cellular longevity, or interaction with antigens or fragments/epitopes thereof presented by select antigen presenting cells, such as follicular dendritic cells or long-term memory B cells. However, much remains to be elucidated regarding the mechanisms that underlie induction and maintenance of immunological memory through intrinsic or extrinsic pathways following infection or vaccination. In addition, there is sufficient evidence to strongly suggest important differences in induction and maintenance of memory in mucosal inductive and effector sites compared to systemic sites. In addition, the rules that dictate the fate of memory TH, CTL, and B cells appear to be differentiated divergently.

In this special issue, we had important contributions in most aspects of B and T cell memory of both mucosal and systemic origins. The study of the quality of antibodies produced by memory B cells as defined by their higher avidity against specific antigenic epitopes is of paramount importance. In this regard, Krueger et al. reported the existence of a recently described plasma cell population that originated from memory B cells, lived in bone marrow, and secondary lymphoid organs, rapidly produced higher avidity antibodies than primary plasma cells, but was short-lived. The same main authors reported another important discovery (Krueger et al.) that involved the essential role of TLR signaling, and hence innate responses, in the induction of the aforementioned memory B cell derived secondary plasma cells. The interplay of innate and adaptive responses in the maintenance of memory B cells has been studied and debated for some time. In 2006, Nemazee's team reported that MyD88-deficient mice were still able to respond normally to LPS-derived, as well as non-TLR-related, but inflammasome-inducing, vaccine adjuvants, such as Alum, hence suggesting that TLR signaling was not a pre-requisite for B cell responses using such adjuvants (1).

Currently, vaccine schedules in humans, and even in animal models, are generally based on what schedule induces high enough acute responses, and it is not known which vaccination schedule induces the highest acute and memory responses. Therefore, the article by Mantile et al. was of high consequence as it identified a “consolidation phase” in the induction of epitope-specific memory B cells, as a window of time during which certain disrupting stimuli could hamper the generation of memory. This phenomenon, if confirmed by others, could have significant consequences on the vaccination schedules.

The issue of memory induction following vaccination is linked to vaccine adjuvants and delivery systems, and their nature as replicating, metabolically alive but non-replicating, or non-living and non-replicating (such as TLR agonists, Alum, etc.) The commonly unwritten rule is that vaccine adjuvants and delivery systems must induce pro-inflammatory innate responses in order to induce acute and memory responses, although this view has recently been challenged by the introduction of a vaccine adjuvant that induced strong adaptive B and T cell responses in the absence of strong innate immune responses (2, 3).

Whether memory cells reside locally in tissues, particularly in mucosal tissues which are the main portal of pathogen and antigen entry, or are derived from other peripheral sites, has been another topic of paramount significance in memory maintenance. Two original papers in this issue addressed this very problem elegantly. In an influenza A model of infection, Suarez-Ramirez et al., showed the significance of the mucosal homing receptor CD103 (α_E_β7) expression that can define tissue resident memory and primary CD8+ CTL in lungs and respiratory draining lymph nodes (LN), the mediastinal LN. Moreover, the check point inhibitor PD-1 and TGFβ also played differential roles on the primary/naïve and resident antigen-experienced CTL. As for the role of resident CD4+ memory TH cells, in a *Helicobacter pylori* model of infection, Liu et al. demonstrated the importance of local subserous vs. traditional remote vaccine administration for induction and maintenance of memory tissue resident CD4+ TH cells in the stomach, and these vaccine induced T_RM_ CD4+ cells played a main role in protection against *H. pylori* infection upon challenge. Because CD4+ TH cells, which recognize more diverse epitopes on influenza viruses than B cells, play a central role in B cell memory responses, Nelson and Sant devoted their paper on how CD4+ T cell imprinting and editing impacts overall memory B cell responses in humans against influenza virus infections.

Much of what we have inferred about the induction and maintenance of immunological memory in humans has been from animal and mostly murine studies. Therefore, the review by Palm and Henry was valuable for shedding light on the differences and similarities between B cell memory induction and maintenance following vaccination and infection in humans and mice. Influenza infections in humans and how to prevent them through induction of memory B cell responses that can be neutralize multiple seasonal or pandemic strains, was the focal point of the review by Auladell et al. with co-authorship by Dr. Peter Doherty, a Nobel prize winner, who has recently focused also on influenza vaccines. In this review, the role of B and follicular T cells in memory induction was examined. Special attention was also placed on the potential of inducing memory CTL responses against conserved regions of influenza viruses in the context of influenza infections and vaccinations, while bearing in mind that differences in HLA types may hamper such efforts in vaccine development. In their Perspective Article, Takamura and Kohlmeier highlighted the differences in T_RM_ vs. circulating lung CD8+ T cells, in the context of central and T_EM_ cells, and delineated special niches within lungs that mainly contain T_RM_. Understanding how and which exogenous cytokines influence memory cells is highly valuable and in this context Kalia and Sarkar pointed out that IL-2 plays a key role in maintaining effector and memory CD8+ T cells, by triggering metabolic and transcriptional alterations in such cells.

Overall, this issue addressed many central questions, but many more remain unanswered on the path to understand and employ various memory stages and processes in vaccine development and vaccination schedules. Methods of tagging epitope-specific cells and tracking them throughout the evolution of acute vs. memory responses from first contact with the epitope until death with regards to their transcriptional signature at various stages, and their interactions with the antigen/pathogen as well as with other cells and cellular products could shed much needed light on the enigma of long-term immunological memory. Specifically, tagging epitope-specific IgM+ B cells throughout their lifetime, and to determine how some acquire many mutations, without switching to downstream isotypes such as IgG, IgA, or IgE, leading to secreted IgM with higher affinities, and whether and when they traffic to the bone marrow, may be a good start on this long path. Such studies may identify hitherto unknown ways of considering the concept of immunological memory (4).

## Author Contributions

The author confirms being the sole contributor of this work and has approved it for publication.

### Conflict of Interest

MV was employed by the company EpitoGenesis, Inc.
